# When fixing problems kills personal development: fMRI reveals conflict between Real and Ideal selves

**DOI:** 10.3389/fnhum.2023.1128209

**Published:** 2023-08-03

**Authors:** Anthony Ian Jack, Angela M. Passarelli, Richard Eleftherios Boyatzis

**Affiliations:** ^1^Philosophy, Psychology, Neurology, Neuroscience, Organizational Behavior Case Western Reserve University, Cleveland, OH, United States; ^2^Coaching Research Lab, Weatherhead School of Management, Case Western Reserve University, Cleveland, OH, United States; ^3^Management and Marketing College of Charleston, Charleston, SC, United States; ^4^Organizational Behavior, Psychology, Cognitive Science Weatherhead School of Management, Case Western Reserve University, Cleveland, OH, United States

**Keywords:** fMRI, intentional change theory, self-knowledge, coaching, behavior change, self-acceptance and growth

## Abstract

Many coaching approaches aim to change behavior by increasing self-knowledge. However, self-knowledge can be difficult to achieve. One hypothesis (e.g., Jung, Rogers) is that self-knowledge is challenging because there is inherent conflict between different aspects of the self. This hypothesis is foundational to Boyatzis’ intentional change theory (ICT). ICT holds that effective coaching requires deliberate sequencing of the client’s exploration of different aspects of their self. Coaches initially encourage clients to focus exclusively on their Ideal self. The ICT approach differs from that advocated by most coaching organizations that suggest collaborative goal setting at the start of the coaching engagement, encouraging clients to focus on fixing performance deficits and problematic behaviors–aspects of the Real self. If there is conflict between thinking about Ideal and Real selves, then this strategy will be suboptimal. The hypothesis of attentional conflict therefore has significant implications for coaching practice. Previous findings establish a link between attention to Ideal vs. Real selves and global vs. local visual processing, respectively. This association alone does not imply conflict because, in naturalistic settings, global and local perceptual processes usually work in concert. However, certain stimuli such as Navon figures (letters made from many smaller letters, e.g., a large E made of small R’s) create conflict due to incongruence between the global and local features. Does thinking about the self inherently generate conflict, like a Navon figure, or is it more akin to everyday perception? To answer this question the current study uses functional magnetic resonance imaging to examine the overlap in brain activity in young adults between two pairs of otherwise very dissimilar tasks: coaching interactions focused on Ideal vs. Real self; and attention to global vs. local features of Navon figures. Despite the ostensible absence of overlap in the psychological processes involved in these pairs of tasks, we find a remarkable degree of overlap in brain activity. This overlap was pronounced in higher (parietal and temporal) areas known to be involved in resolving attentional conflict. These findings provide compelling biological evidence for inherent conflict between thinking about Ideal and Real selves.

## 1. Introduction

Coaching is a rapidly expanding development activity in many organizations. Professional coaches are increasingly hired to support the development of valued employees. The coach’s role in such interventions is to inspire self-directed learning and persistence in one’s efforts to develop, resulting in sustained positive change (Bono et al., 2009; Athanasopoulou and Dopson, 2018). Yet sustained change–particularly behavior change–is notoriously difficult to achieve. There is broad agreement among coaching scholars that enhanced self-knowledge is critical to change (Boyatzis et al., 2015; Yip et al., 2020; Bachkirova, 2022). Moreover, there is general agreement that the self is complex and multifaceted (Markus and Nurius, 1986; Higgins, 1987; Ibarra, 1999). The multifaceted nature of the self is central to intentional change theory (ICT, Boyatzis, 2006), which holds that sustained change is predicated on acceptance of an aspirational “ideal self” that is later contrasted to a more current “real self” in a complex discovery process. Research on ICT-based coaching interventions indicates that this bears out in practice (Boyatzis et al., 2002; Van Oosten et al., 2019; Passarelli et al., 2022).

Despite evidence that purposeful sequencing of discussion of different facets of self is critical to sustained change, it has been slow to take hold in coaching practice. Many associations, certification agencies, and training groups recommend focusing a client’s attention on their immediate goals or problems early in the coaching engagement as standard practice. In so doing, the coach focuses a client’s attention on their Real self and suppresses consideration of their “bigger picture”–the personal vision, dream and sense of purpose associated with the Ideal self. As a result, they miss engaging the psychophysiological state that is most conducive to sustained effort at learning and change (Jack et al., 2013; Howard, 2015; Passarelli, 2015).

At the heart of this problem of practice is lack of theory to adequately address the complexity of the self. Is it possible to attend to both the Ideal self and Real self simultaneously? Or does the salience of the Real self interfere with the accessibility of the Ideal self? This study extends prior research (Jack et al., 2013) to directly address the question of conflict between different aspects of the self. Psychology has long recognized parallels between the social and emotional processes involved in personal development on the one hand, and visual perception on the other. These parallels motivated the development of Gestalt therapy (Perls et al., 1951), which in turn grew out of Gestalt psychology, a school that emphasized the importance of holistic and meaning-making processes in perception. Our previous neuroimaging study revealed a concrete basis for the metaphorical links between personal development and visual perception by showing that brain regions traditionally thought to be specialized for visual processing are also engaged during effective coaching. The current study explores those links in greater depth by examining overlap in neural activity between two pairs of apparently unrelated tasks: coaching to the Ideal versus Real self, and visual attention to global versus local perceptual features.

Our study makes several contributions to the literature. First, we advance intentional change theory (ICT) and our understanding of possible selves in general by introducing the concept of acceptance. Second, our findings provide support for the hypothesis that conflict sometimes arises between Ideal and Real selves. Finally, we offer recommendations for coaching practice based on these findings.

## 2. Γνωθει σ’αυτ*o*ν (know thyself)

The belief that self-knowledge holds the key to living a good life is a recurring theme in Western thought. The ancient Greek aphorism “know thyself” was the first of three maxims inscribed at the Temple of Apollo at Delphi and guided the thinking of many of the ancient philosophers (Laertius, 225 CE, White, 2021). More recently, with the emergence of psychology as a discipline distinct from philosophy, some influential thinkers began to address the question of why self-knowledge is often difficult to acquire. Carl Jung viewed integration of “persona” and “shadow” as one of the main tasks of human development. Jung held that healthy development requires that this integration be led by the persona rather than the shadow (Jung et al., 1953). He also emphasized that the shadow should ultimately be embraced rather than rejected. Carl Rogers was perhaps the most influential proponent of humanistic psychology, in contrast to the behaviorist and psycho-analytic approaches that preceded it (Rogers, 1970). Rogers thought that everyone experiences incongruence between their “Ideal” and “Real” selves. To help lessen this conflict, Rogers emphasized a client-centered approach with empathic listening used as a key tool to support the individual’s innate drive for self-realization.

Roger’s approach stands in contrast to the problem-fixing orientation more familiar in psychology, which draws strongly on the medical model of diagnosis and “cure” or treatment. Behavioral Therapy and Cognitive Behavioral Therapy (CBT) are focused exclusively on identifying then fixing problematic behaviors and thoughts. More recently Roger’s central insight has resurfaced in the so-called “third wave” cognitive behavioral approaches. Dialectical Behavior Therapy (DBT) is the best-known of these (Linehan and Wilks, 2015). DBT’s central idea is the need to maintain a balance between change and acceptance. To help foster acceptance, DBT emphases the importance of the therapist adopting a warm and open interpersonal style. Another third wave cognitive behavioral approach is Acceptance and Commitment Therapy (ACT) which similarly recognizes the importance of circumventing psychological resistance to change by placing an emphasis on cultivating acceptance and “willingness” in contrast to “willfulness” (Hayes et al., 2006). Gestalt Therapy, a humanistic school of psychotherapy developed in the 1950’s that remains widely practiced, also emphasizes the importance for change of non-judgmental self-awareness (Perls et al., 1951). Gestalt’s Paradox of Change is the idea that the more we accept ourselves as we are right now, the more it becomes possible for us to change.

Many contemporary schools of coaching also view the cultivation of self-knowledge as central. Coaches often use stories about the self to stimulate change (Taylor et al., 2019; Yip et al., 2020). The self is multifaceted in nature (Markus and Nurius, 1986; Higgins, 1987; Ibarra, 1999). Hence, the self-narratives evoked in coaching conversations tend to focus on different salient aspects of the self. Two aspects of the self are most relevant to coaching. Visioning work helps the person being coached articulate the story of a desired future and evokes a narrative about one’s Ideal self (Boyatzis and Akrivou, 2006; Taylor et al., 2019). Ideal self-narratives answer the question, who do I want to be? In contrast, coaching conversations that begin with evaluation, such as reviewing multisource feedback, evoke a narrative about the Real self (Taylor, 2006). Real self-narratives answer the question, who am I today? Although both self-narratives play essential roles in any change process, there may be an unintended cost when coaches seek to establish the immediate utility of coaching by focusing first and foremost on fixing current problems. This directs the coaching conversation to discussion of the Real self.

## 3. Concepts of self in intentional change theory

**FIGURE 1:**
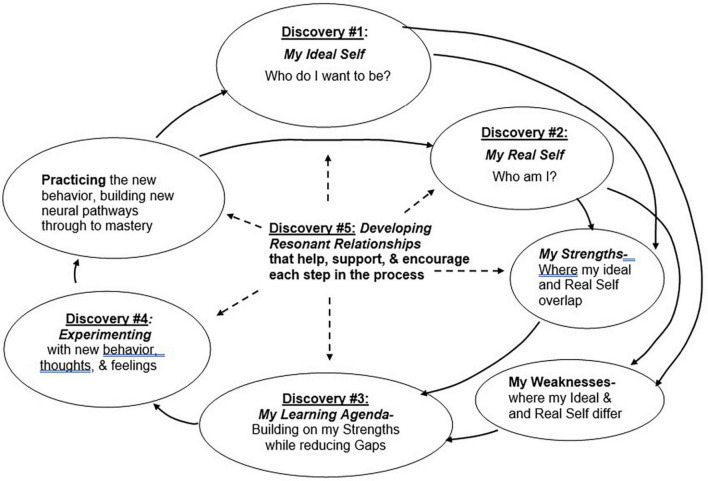
The ICT coaching process involves an ordered sequence of discoveries. The first and most important discovery involves the exploration of the ideal self (Boyatzis, 2006, 2008).

**FIGURE 2:**
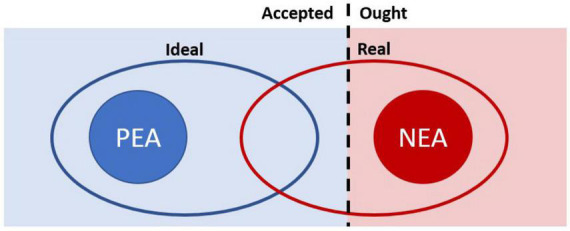
Different aspects of the self and relationship to the two hypothesized psychophysiological states evoked by the two coaching conditions in the current experiment.

According to intentional change theory (ICT, Boyatzis, 2008), sustained development occurs through a complex process in which individuals adopt new behaviors to approach their Ideal self or reduce the discrepancy between an Ideal self and Real self (Higgins, 1987; Boyatzis, 2008). The process of intentional change (Figure 1) involves five discoveries: (1) articulation of the ideal self, (2) increased awareness of the real self, (3) setting a learning agenda to move toward one’s ideal self, (4) implementing the learning agenda through deliberate action, and (5) leveraging supportive relationships for one’s change efforts. ICT views the conflict between different ways of thinking about the self as a fundamental tension that forms the foundation of the ICT approach to coaching. ICT builds on key insights of Jung, Rogers and others and elaborates them in two ways: first it offers a more thorough theoretical explanation of the underlying conflict based on current research in neuroscience and psychology; and second it prescribes a concrete and actionable sequence for effective coaching based on this theoretical model. Here we additionally update ICT theory to incorporate a more nuanced account of different aspects of the self (Figure 2). Prior theorizing in ICT acknowledges the ought self, but until now has not explicitly articulated the role of acceptance in promoting integration of different aspects of the self (Ideal and Real). This update to ICT aligns with theorizing on “radical acceptance” and “willingness,” key concepts in Dialectical Behavior Therapy and Acceptance and Commitment Therapy, respectively.

Here we focus on four distinct aspects of self, which may be briefly defined as follows:

• **Ideal self:** the individual as they most desire to be, with their strengths fully realized and their weakness minimized.
• **Real self:** the individual as they are presently showing up, including both strengths and weaknesses.
• **Accepted self:** aspects of the self that the individual embraces and accepts.
• **Ought self:** aspects of the self, often externally imposed, that the individual judges and rejects.

Intentional change theory holds that thinking about aspects of the self that are both Ideal and Accepted elicits a psycho-physiological state called the Positive Emotional Attractor (PEA). By contrast, thinking about aspects of the self that are both Real and Ought elicits a psycho-physiological state called the Negative Emotional Attractor (NEA). Critically, the PEA and NEA are held to be mutually incompatible states around which cognition and behavior related to personal development are organized. Table 1 provides a fuller list of the contrasting characteristics of the two attractors.

**TABLE 1:** Characteristics of the two psycho-physiological attractors.

	Positive emotional attractor	Negative emotional attractor
Primary self focus	Ideal and accepted self	Real and ought self
Autonomic Nervous System	Parasympathetic arousal	Sympathetic arousal
Cortical networks	Empathic Network aka “Default Mode Network”	Analytic Network aka “Task Positive Network”
Affective valence	Positive	Negative
Cognitive-affective state	optimism, hope	pessimism, fear
Self Determination Theory	Intrinsic motivation	Extrinsic motivation
Self-regulatory focus	Promotion: attaining gains	Prevention: avoiding losses
Orientation	Possibilities, dreams	Problems, expectations
Focal Capabilities	Strengths	Weaknesses
Learning agenda actions	Self-generated experiments	Compliance behaviors
Practice	Practice to mastery	Practice to comfort
Relationship tone	Resonant/Inspiring	Dissonant/Annoying

The ICT coaching process (Figure 1) is hypothesized to act on different aspects of the self as follows:

(1) The first discovery increases the salience and clarity of the Ideal and Accepted self. This provides a powerful motivational anchor and gives the coachee a basis for generating their own goals rather than following external expectations.
(2) The second discovery leverages the increase in self-acceptance achieved by the first discovery to allow the coachee to acknowledge and accept more of their Real self. The result is an increase in overlap between Real and Accepted aspects of self. This engenders willingness to change instead of the psychological resistance, denial, and willfulness engendered by the Ought self. As a result, the coachee becomes more open to feedback and processes it more effectively.
(3) The third, fourth and fifth discoveries, and engaging in the ongoing cycle, gradually bring the Real self closer to the Ideal self, increasing overlap between Real and Ideal selves. In addition, as the coachee experiences how personal development can be more effectively intrinsically motivated by a vision of their Ideal self (rather than extrinsically motivated by external expectations), the salience of their Ought self decreases. This increases their openness to continued personal development.

In terms of how to practically approach coaching, ICT outlines a sequence of “discoveries” through which the coach guides the coachee (Boyatzis, 2008). This sequence is outlined in Figure 1. Most significant for current purposes is that ICT emphasizes that the first (and the most important) discovery is the client’s elaboration of their Ideal (and Accepted) self. This is encouraged through questions and exercises that help the coachee to identify their key values, passions, purpose, and strengths. The coachee is also encouraged to envision an ideal future that can serve as a motivational anchor for their personal development. Importantly, the emphasis during this first discovery phase of the coaching explicitly excludes any assessment of current performance and/or problems or any consideration of organizational expectations. Only after the first discovery has been well elaborated and thoroughly felt is the coachee encouraged to explore their Real self, for instance by seeking feedback from others via multisource assessment. This elaboration of the Real self is viewed strictly through the lens of serving the coachee’s goal to move closer to their Ideal self. This approach addresses the concern that feedback is often found to be deleterious to performance rather than performance enhancing (Kluger and DeNisi, 1996). It is corroborated by the empirical finding that individuals are better able to effectively incorporate feedback if they are first primed to think about their personal values (Falk et al., 2015). Another important aspect of ICT is how the second discovery (exploration of the Real self) anticipates the third discovery (the learning agenda). Here the emphasis is placed firmly on building on the individuals’ strengths, with a reduced emphasis on addressing weaknesses.

Careful framing of the exploration of the Real self, so it is (i) seen as being in service of the Ideal self, and (ii) focused on developing strengths, are two critically important features of ICT because they foster greater acceptance of the Real self. As mentioned earlier, the NEA is associated with thinking about the Real and Ought self. Thinking about the Real and Accepted self does not cause the coachee to enter the NEA. By increasing acceptance, this framing minimizes conflict between the Ideal and Real selves. By contrast, the way that performance reviews are usually conducted in organizations evokes the NEA because the focus is usually put on externally imposed expectations that highlight Ought aspects of the Real self. Similarly, starting a coaching conversation by focusing on concrete goals directs attention to Ought aspects of the Real self, and so evokes the NEA rather than the PEA. For these reasons, ICT holds that it is very important to sequence coaching conversations by focusing first and foremost on the client’s Ideal and Accepted self. Even though the coach may be otherwise highly competent and the coachee sincerely motivated to change, ICT predicts that coaching approaches that frame the change process in terms of externally defined goals are likely to have the unintended consequence of inhibiting the individual’s efforts to adapt, grow, or change (Boyatzis et al., 2015).

In addition to ICT three other well-known theories similarly suggest that placing emphasis on the Ideal and Accepted self will be more effective at motivating sustained behavior change than placing emphasis on the Real and Ought self, due to links with positive vs. negative affect, intrinsic vs. extrinsic motivation, and promotion vs. prevention motivation (Higgins, 1997; Fredrickson, 2001; Ryan and Deci, 2002). However, unlike ICT, these three theories do not hypothesize conflict between the psychological processes involved. In practical terms, these theories leave open the alternate strategies of interweaving discussions of different aspects of the self or starting with the Real and Ought self. Hence, if the hypothesis of conflict from ICT is borne out, this would have profound implications for coaching theory and practice. It would also provide an explanation for why behavior change has historically proven so challenging. At first sight it appears the most effective way to engender behavior change ought to involve simultaneously harnessing different forms of motivation i.e., using both carrot and stick. However, the hypothesis of conflict between different aspects of the self implies that this seemingly reasonable strategy would have the counterintuitive effect of inhibiting personal development.

## 4. Testing the hypothesis

What evidence might tell for or against the hypothesis of conflict between thinking about the Ideal and Accepted and thinking about Real and Ought selves? Behavioral investigations of social cognition are challenging to control because of the inherent trade-off between ecological validity and the use of precisely quantifiable stimuli and tasks (Schilbach, 2015). Neuroimaging offers a window onto the physiological processes evoked by different psychological conditions. This can be highly informative about the underlying mechanisms that guide our thinking, provided that appropriate care is taken not to overinterpret findings (Jack et al., 2017). Neuroimaging promises to be particularly informative about the hypothesis of conflict between different aspects of the self for two reasons: First, several studies have identified neural signatures associated with resolving conflict between global and local perception. Second, there is good evidence for links between global versus local perception and thinking about Ideal and Accepted versus Real and Ought selves (Förster and Higgins, 2005; Jack et al., 2013; these links are discussed in greater detail in “7. Discussion”).

Neuropsychological and neuroimaging studies indicate that distinct brain areas are specialized for the perception of global versus local visual features. Neuropsychological studies, i.e., studies of individuals with brain lesions, lack anatomical precision because brain damage often impacts the function of nearby tissue. Neuropsychological studies have thus traditionally focused on relatively crude distinctions such as whether the lesion impacts the left or right hemisphere and is anterior (i.e., frontal lobe) or posterior (i.e., occipital, temporal and parietal lobes). These studies have found that individuals with right posterior lesions have impaired global processing, whereas individuals with left posterior lesions have difficulty processing local information (Robertson et al., 1988; Lamb et al., 1989). Neuroimaging studies have corroborated a tendency toward lateralization and have also identified distinct regions in posterior cortex that are preferentially involved in global and local visual processing (Fink et al., 1996, 1997; Martinez et al., 1997; Han et al., 2002, 2004; Weissman and Woldorff, 2005). Both behavioral and neuroimaging evidence indicate that the reliance on different brain areas for global and local processing is attenuated when conflict is low and pronounced when conflict is high (Fink et al., 1996; Hübner and Malinowski, 2002; Weissman and Woldorff, 2005).

**FIGURE 3:**
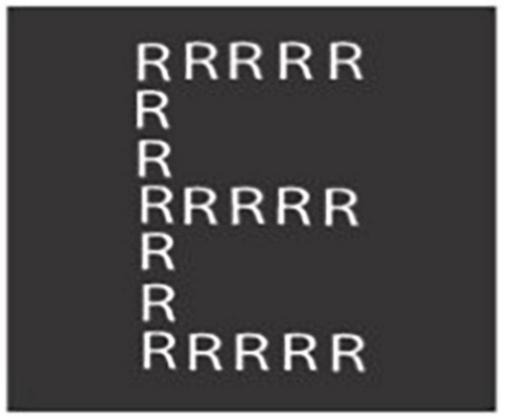
One of the Navon figures used in the visual attention task (local cue).

A type of stimulus often used in studies of global and local perception is a hierarchical or Navon figure (Navon, 1977). This involves a large letter comprised of numerous smaller letters. Figure 3 shows one of the stimuli used in the current study, a large E made of small R’s. Navon figures place specific and unusual demands on the perceptual system. In typical perception, there is little conflict between global and local perception so these processes tend to work in concert. By contrast, the incongruent global and local features of Navon figures create conflict. The rapid and accurate perception of the global (or local) features of such stimuli requires attentional processes to amplify global (or local) information while also minimizing interference by inhibiting local (or global) information.

The current study used fMRI to investigate neural signatures associated with two pairs of otherwise highly dissimilar tasks: two visual attention tasks in which participants were required to respond to either the global or local features of a series of Navon figures; and two tasks involving simulated coaching interactions which led participants to think about either their Ideal and Accepted self or their Real and Ought self. The latter two tasks were closely modeled on our prior fMRI study which provided evidence for a link between thinking about the Ideal self and global perception (Jack et al., 2013). The purpose of comparing brain activity between these two pairs of tasks was to assess the degree of conflict involved in thinking about different aspects of the self. If those processing demands are more akin to those involved in regular everyday perception, where global and local processing work in concert, then there would be no reason to predict similar patterns of brain activity to those observed while processing Navon figures, where attentional processes are engaged to resolve conflict. On the other hand, as anticipated by Jung and Rogers, it might be that when we pause to think about who we are, doing so requires us to resolve inherent conflict between different aspects of the self that spontaneously come to mind and vie for our attention. More specifically, thinking about the Ideal and Accepted self (aka the “persona”) might require us to actively amplify those aspects of the self while minimizing interference by inhibiting thoughts about Real and Ought aspects of the self (aka the “shadow”); and vice versa for thinking about the Real and Ought aspects of self. If this is the case, then we would expect to see more substantial overlap with the patterns of brain activity evoked by the visual attention tasks.

## 5. Materials and methods

### 5.1. Subjects

Forty-seven full time undergraduates participated in this study. An online pre-screen and scheduling tool (Experiment Management System, Sona Systems) was used to establish that subjects were right-hand dominant, native English speakers, had no neurological disorders or diseases, were not pregnant, did not experience claustrophobia, and were not declared Cognitive Science majors. Of the 65 who volunteered for the entire study of which this was a part, 14 were dropped due to high scores on the Depression, Anxiety, and Stress Scale (DASS-21), failing to attend sessions, claustrophobia, or previously undisclosed neurological disorders. fMRI data were obtained from a final total of 51 subjects (26 male, average age = 19.8 years). Four participants had incomplete imaging data, yielding a final sample size of 47 subjects. Subjects were compensated for their time at a rate of $10 to complete an initial questionnaire, and an additional $50 as well as a copy of a leadership book and a structural image of their brain if they completed the remaining study requirements. This research was approved by the appropriate local Institutional Review Board, and informed consent was obtained from all participants.

### 5.2. Design

Our hypotheses were tested using functional magnetic resonance imaging (fMRI) to quantify neural activity while subjects engaged in coaching and visual attention tasks. Consistent with Jack et al. (2013), face-to-face coaching sessions were conducted prior to the fMRI scan. All subjects had one coaching session focused on the real self and were randomly assigned to a varying number of ideal-self coaching sessions (zero, one, two, or three face-to-face sessions or an asynchronous written session). By varying the levels of the ideal-self treatment, we expanded previous work by disentangling prior coaching from scanner arousal. Levels of the ideal-self treatment did not influence arousal in the regions we describe here - hence, these arousals can be attributed to the video-simulated interactions in the scanner task alone.

Coaching conditions were kept consistent between the face-to-face and video-simulated scanner interactions. That is, the same coach who led the real self session appeared in the scanner video making Real self related statements. The coaches were counterbalanced across conditions and gender to minimize coach-specific or gender effects. Coaches were randomly assigned to the Ideal Self or the Real Self condition for each subject. In order to standardize timing, all Ideal self sessions were conducted first and within 1 week of each other (in multiple session conditions), followed by the Real self session. fMRI scans were conducted within 2 weeks of the final session.

Face-to-face coaching session(s). Two experienced coaches of different genders were trained in the study protocol to conduct semi-structured coaching sessions. After introducing himself/herself, the coach in the Ideal self condition asked the anchor question: “If everything worked out ideally in your life, what would you be doing in 10 years?” This was followed with more specific probes if needed. In addition to this theme of vision for the future, subjects who were assigned to two or three Ideal self sessions also discussed (2) compassion via people who have helped the student most in the past, and (3) mindfulness with a discussion of their current most important values.

In the Real self condition, the coach asked the anchor question: “What challenges have you encountered or do you expect to encounter in your experience here?” If needed, additional probes were used. The aim of the Real self session was to focus the participant on assessing their current performance and anticipate the future in terms of what they should be doing to achieve what they believed was expected of them. The coaches always maintained a polite and respectful tone.

### 5.3. Neuroimaging sequence

In the scanner, each participant received seven scans (MR acquisition sequences). The first two scans established the unique anatomical structure of that individual’s brain and allowed the functional data to be transformed to a standardized atlas space (711-2B, with coordinates approximating standard Talairach space). The other five scans were functional scans, used to assess BOLD response to different task conditions. The first functional scan is not of relevance here, and involved participants viewing pictures of static faces. The next three functional scans required participants to engage in the simulated coaching task. The final functional scan required participants to engage in the visual attention task.

### 5.4. Coaching task

The coaching task simulated Ideal and Real self-based coaching interactions in a video-conference-style interaction between the participant and the coaches. The participant was presented with a total of 96 pre-recorded videos of the coaches making statements about the participant’s educational experience or outlook on the future. These statements were developed around the themes of hope, compassion, mindfulness and playfulness in the Ideal self condition and lack thereof in the Real self condition (e.g., ideal self: “I am excited about the possibilities my future holds,” and real self: “I am afraid I will not achieve what is expected of me”). Equal numbers of present and future-focused statements were included. Using a button press, subjects indicated the degree to which they agreed or disagreed with the statement on a four-point scale (1 = strongly disagree, 4 = strongly agree). As soon as the subject responded, a brief video clip played in which the coach thanked the participant for their response. Hence, each trial involved a pseudo-interactive exchange between the participant and one of the two interviewers.

**FIGURE 4:**
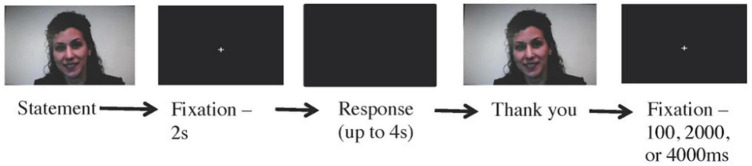
Coaching task sequence.

The timing of an individual trial of the coaching task is depicted in Figure 4. The videos consisted of a face-on view of the head and shoulders of the coach, who maintained eye contact (i.e., looked at the camera) as s/he spoke. Each coach made statements in a tone congruent with the style of coaching session previously experienced by the participant with that coach (mean length = 5193.54 ms), followed by a 2-s fixation cross. Then participants had 4 s to respond to the statement. After the response or the passing of 4 s, a short video of the interviewer thanking them for their response played (mean length = 2218.34 ms). A fixation of 100 ms, 2,000 ms, or 4,000 ms was randomly inserted between each trial. Participants underwent three fMRI runs of this task for 280 frames or 560 s. Each fMRI run comprised 32 experimental trials (16 of each condition, ideal and real self) and 8 resting fixation periods of 15 s.

### 5.5. Visual attention task

A stimulus from the visual attention task is depicted in Figure 3. The visual attention task consisted of a cued-version of the Navon (1977) figures task, which was used to localize brain regions involved in resolving conflict between global and local perceptual features. Participants were instructed to look for either the large (global) or component (local) letters in blocks of 10 Navon figures. The large letters were either (1) R’s and L’s comprised of smaller letters (e.g., A, H, X) or (2) other letters (A, H, X) made up of small R’s and L’s. Participants responded to seeing the letter “R” with their right hand and the letter “L” with their left hand. One second was allotted to respond to each letter. If the participant responded before 1 s passed, the slide switched to a fixation cross for the remaining time. The first two blocks of each type were considered practice. This task took 280 frames (whole volume functional acquisitions), or 560 s.

### 5.6. MRI Image acquisition

A 4-Tesla Siemens-Bruker hybrid research MRI scanner was used. Participants experienced structural image acquisition (T1 and T2w), and five blood oxygenation level-dependent (BOLD) runs (TR = 2,000, TE = 20, flip angle = 90°) containing 310 or 280 echo planar imaging (EPI) volumes each (one of these runs was for the purpose of a different study). BOLD EPI images consisted of 38 contiguous 3.8 mm slices, producing 3.8 mm cubic voxels. The experimental paradigm, presented using E-Prime 2.0 software, was projected onto a screen on the head coil and visible through an angled mirror adjusted to accommodate each participant. Responses from four buttons, two under the index and middle fingers of each hand, were recorded and time stamped by E-Prime 2.0.

Preprocessing was accomplished using software developed, tested, and maintained by Abraham Snyder at Washington University in St. Louis Medical School.^1^ Dr. Synder consulted on the analysis. Motion was corrected across and within runs using a rigid-body rotation and translational algorithm. Spatial transforms were computed to realign BOLD images where they were moved into a common atlas space determined by co-registration of an average EPI image with the T2 weighted structural image. The T2 weighted structural image was aligned with the T1 weighted magnetization-prepared rapid-acquisition gradient echo (MP-RAGE) structural sequence. The T1 image was aligned with an average T1 atlas through a series of affine transformations. This atlas had been created specifically for the Bruker 4T magnet and was aligned to the Washington University in St. Louis 711-2B version of Talairach atlas space (Buckner et al., 2004). The resulting atlas represented Talairach space according to the SN method (Lancaster et al., 1995). The BOLD images were then re-sampled into 3 mm isotropic voxels. Data was smoothed using a 2 voxel (6 mm) FWHM Gaussian kernel. Subsequent analyses of BOLD data used a general linear model (GLM), modeling out baseline and linear trends, and assuming a standard hemodynamic response function.

### 5.7. Imaging data analysis

All reported findings are based on quantitative analysis and established inferential statistics using the Washington University in St. Louis software, FIDL, created and maintained by Dr. Mark McAvoy.

Data for each participant was entered into a general linear model in which baseline and linear trend were estimated alongside an assumed hemodynamic response function (HRF) associated with each condition. The assumed hemodynamic response function used was similar to that used by a variety of common neuroimaging analysis programs and is based on careful work modeling the HRF to visual stimuli in V1 (Boynton et al., 1996). The coaching task and analysis relied on an extended event design (i.e., an event which was long in time). For the sake of the analysis presented, we collapsed activity across the three phases of each event (video, response, and thank you–see Figure 4). For the visual attention task, a blocked analysis and design was used (i.e., participants experienced blocks of trials in which they were required to identify either local or global letters throughout the block). The task design also allowed for event-related analyses of the visual attention task (i.e., there were random jitters in times between trials in the block). This analysis was also conducted and produced similar but statistically weaker findings than the block design analysis.

Statistical inference was made based on random effects analyses, in which each participant contributed one estimate for each condition derived from the individual participant GLMs. The degrees of freedom for these analyses were determined by the number of participants. The contrasts used in the cognitive conjunction analysis were derived using paired *t*-tests.

We used cognitive conjunction analysis, which is an inferential technique for establishing that a brain region is activated by two statistically independent comparisons (Price and Friston, 1997; Friston et al., 2005). For the cognitive conjunction analysis, two contrasts were first calculated. One contrast was between the two coaching tasks (ideal–real), the other between the visual attention tasks (global–local). Next, each contrast was separated into two images, corresponding to positive (e.g., ideal > real, and global > local) and negative (e.g., real > ideal, and local > global) contrast values. Each of these images contained only positive values corresponding to the absolute value (modulus) of the contrast difference, with all other values set to zero. These images were then combined in pairs to form conjunction images by taking the minimum z-score at each voxel for the two images (i.e., the value assigned to each voxel is the lesser of the value revealed by the two contrasts). In other words, the conjunction image of real/global was set to the minimum value of the pair | real–ideal | and | global–local |, but only for those voxels where real–ideal > 0 and global–local > 0. All other voxels were set to zero. It followed as a matter of mathematical necessity that most of the voxels in the conjunction images were set to zero. The ideal/local and real/global conjunction images comprised very few non-zero voxels, whereas the ideal/global and real/local conjunctions did contain several non-zero voxels that reflected the hypothesized associations (see “6Results”).

All reported findings were corrected for whole brain multiple comparison correction using a threshold and clustering criterion (Threshold z = > 3, Cluster: number of contiguous voxels = > 17). These thresholds were established through monte-carlo simulations run on real fMRI data (McAvoy et al., 2001). In standard cognitive conjunction (Price and Friston, 1997), the threshold for multiple comparison correction is decreased (divided by root 2, so making it more lenient) to reflect the increased probability of Type II error while holding Type 1 error at a reasonable level. In strict cognitive conjunction (Nichols et al., 2005), the threshold for multiple comparison correction is not altered, allowing a stronger inference (i.e., researchers can state that they have independently established that the region is independently implicated by each of the contrasts). However, strict cognitive conjunction is a highly conservative statistical test prone to Type II error (Friston et al., 2005). Since there has been some controversy about which threshold is most appropriate, we report both findings. Notably, most areas identified by the standard cognitive conjunction analysis in our tests also included smaller areas that passed strict cognitive conjunction. For these cases, the issue of Type I error has effectively been ruled out, and the standard cognitive conjunction provides a more reasonable estimate of the extent of the regions which are involved in both contrasts.

## 6. Results

### 6.1. Manipulation check

To check that the coaching tasks were having the expected and desired effect on the subjects, a brief survey was administered right after each coaching session. The items used emotions that were predicted to be aroused according to ICT and the design of the coaching tasks. Results of the manipulation check were in the predicted direction for all participants. In fact, there were significant differences in participants’ perceptions of the Ideal Self and Real Self coaches with regard to the quality of the relationship (the subjects felt more trust, rapport after the PEA sessions as contrasted to the NEA sessions) and the motivational strategy (the subjects felt more inspiration and less obligation after the PEA sessions as contrasted to the NEA sessions) used by the coach, as shown in Table 2.

**TABLE 2:** Manipulation check results.

	Real self coaching		Ideal self coaching		*t*-test
	***M***	**(SD)**	***M***	**(SD)**	
Trust	3.52	(1.5)	6.08	(1.1)	*t*(23) = −6.12*
Rapport	3.49	(1.3)	6.10	(0.9)	*t*(23) = −7.38*
Inspiration	3.22	(1.2)	4.84	(1.0)	*t*(22) = −4.57*
Obligation	5.51	(1.3)	2.30	(1.3)	*t*(23) = 9.39*

*p < 0.001.

### 6.2. Behavioral results

Behavioral results are reported for 46 participants (data was corrupted for one subject). The behavioral results provide evidence of the efficacy of both the visual processing and coaching tasks. For the visual attention task, overall accuracy on all trials was 96.82% (global = 97.94%, local = 95.90%). High accuracy on this task signifies that subjects were successful in their attempts to attend to the global or local features of the image. The average response time for all correct trials was 415.19 ms. As expected on the basis of prior work (Navon, 1977), subjects responded more quickly to global than local trials (*t* = 11.42, *p* < 0.001; Mglobal = 392.43 ms, Mlocal = 434.35 ms).

For the coaching task, subjects agreed overall with statements in the coaching trials more than they disagreed (*M* = 2.90), and agreed with ideal-self coaching statements more than real-self coaching statements (*t* = 13.02, *p* < 0.001, Mideal = 3.35, Mreal = 2.45). Average response time to statements was 656.76 ms, and subjects responded more quickly to ideal self statements than to real self statements (*t* = −3.60, *p* < 0.001, Mideal = 624.99, Mreal = 689.65).

### 6.3. Imaging results

The standard approach to the cognitive conjunction analysis (Price and Friston, 1997) revealed extensive overlap in cortical areas in the occipital (visual) cortex, as well as regions at the temporo-parietal junction and medial parietal cortex, as shown in Table 3. A stricter statistical approach, advocated by some researchers (Nichols et al., 2005) but regarded as overly conservative by others (Friston et al., 2005), revealed eight regions, which were less spatially extensive but included all the key areas identified in the first analysis, as shown in Table 4. Taken together, these results suggest that our primary hypothesis of overlap was confirmed with very little likelihood of either Type I or Type II error.

**TABLE 3:** Overlapping brain regions, standard conjunction analysis.

Location of center of mass	Talairach coordinates (x, y, z)	Extent (voxels)	Condition
Left cerebrum, Occipital lobe, **Fusiform gyrus**, Gray matter, Brodmann area 18, Range = 2	(−20, −89, −9)	812	real/local
Right cerebrum, Occipital Lobe, **Lingual gyrus**, Gray matter, Brodmann area 18, Range = 0	(27, −96, −3)	234	real/local
Right cerebrum, Sub-lobar, **Insula**, Gray matter, Brodmann area 13, Range = 1	(31, 14, −6)	60	real/local
Right cerebrum, Frontal lobe, **Superior frontal gyrus**, Gray matter, Brodmann area 9, Range = 0	(9, 59, 26)	17	real/local
Left cerebrum, Temporal lobe**, Caudate**, Gray matter, Caudate tail, Range = 3	(−35, −38, 6)	150	ideal/global
Right cerebrum, Occipital lobe, **Cuneu**s, Gray matter, Brodmann area 30, Range = 0	(14, −67, 12)	537	ideal/global
Left cerebrum, Occipital lobe, **Middle occipital gyrus**, Gray matter, Brodmann area 19, Range = 4	(−36, −69, 13)	454	ideal/global
Left cerebrum, Sub-lobar, **Claustrum,** Gray matter, Range = 1	(−38, −20, −4)	17	ideal/global
Right cerebrum, Temporal lobe, **Superior temporal gyrus**, Gray matter, Brodmann area 22, Range = 3	(51, −52, 18)	618	ideal/global
Left cerebrum, Frontal lobe, **Middle frontal gyrus**, Gray matter, Brodmann area 8, Range = 3	(−22, 22, 35)	156	ideal/global
Left cerebrum, Sub-lobar, **Insula**, Gray matter, Brodmann area 13, Range = 1	(−37, −12, 13)	17	ideal/global
Right cerebrum, Parietal lobe**, Precuneus**, Gray matter, Brodmann area 7, Range = 1	(1, −48, 49)	247	ideal/global
Left cerebrum, Frontal lobe, **Sub-Gyral,** White matter (No gray matter found within ± 5 mm)	(−35, −18, 31)	35	ideal/global

Location of center of mass includes output from Talairach Client set to search nearest gray matter to given coordinates.

**TABLE 4:** Overlapping brain regions, strict conjunction analysis.

Location of center of mass	Talairach coordinates (x, y, z)	Extent (voxels)	Condition
Left cerebrum, Occipital lobe, **Inferior occipital gyrus**, Gray matter, Brodmann area 17, Range = 1	(−19, −94, −6)	173	real/local
Right cerebrum, Occipital lobe, **Lingual gyrus**, Gray matter, Brodmann area 18, Range = 0	(28, −97, −3)	43	real/local
Right cerebrum, Limbic lobe, **Parahippocampal gyrus**, Gray matter, Brodmann area 19, Range = 1	(17, −49, −5)	26	ideal/global
Right cerebrum, Occipital lobe, **Lingual gyrus**, Gray matter, Brodmann area 18, Range = 0	(11, −68, 1)	27	ideal/global
Right cerebrum, Occipital lobe, **Middle temporal gyrus**, Gray matter, Brodmann area 19, Range = 2	(43, −62, 13)	61	ideal/global
Left cerebrum, Temporal lobe, **Middle temporal gyrus**, Gray matter, Brodmann area 39, Range = 4	(−38, −71, 16)	42	ideal/global
Right cerebrum, Parietal lobe, **Inferior parietal lobule**, Gray matter, Brodmann area 40, Range = 1	(60, −35, 28)	41	ideal/global
Right cerebrum, Parietal lobe, **Precuneus**, Gray matter, Brodmann area 7, Range = 0	(4, −49, 50)	41	ideal/global

Location of center of mass includes output from Talairach Client set to search nearest gray matter to given coordinates.

We also conducted a conjunction analysis in the opposite direction in order to determine the extent of overlap between real-self coaching and global attention as well as ideal-self coaching and local attention. Pairing the task conditions opposite to the predicted direction produced limited results, as shown in Table 5. Using the standard threshold (less strict), 1,031 voxels showed overlap for global-real/local-ideal as compared to 3,469 voxels for global-ideal/local-real. Moreover, there was no overlap at all for global-real/local-ideal using the strict threshold for conjunction analysis. This is further support for one of the hypotheses of this study (i.e., that ideal self coaching is associated with global processing and real self coaching is associated with local processing at the neurobiological level).

**TABLE 5:** Overlapping brain regions, standard conjunction analysis, opposite conjunction to hypothesis.

Location of center of mass	Talairach coordinates (x, y, z)	Extent (voxels)	Condition
Left cerebellum, Posterior lobe, **Pyramis**, Gray matter, Range = 0	(−19, −77, −34)	38	real/global
Left cerebrum, Temporal lobe, **Middle temporal gyrus**, Gray matter, Brodmann area 22, Range = 3	(−52, −40, 0)	203	real/global
Left cerebrum, Frontal lobe, **Inferior frontal gyrus**, Gray matter, Brodmann area 47, Range = 5	(−41, 33, 2)	38	real/global
Right cerebrum, Temporal lobe, **Middle temporal gyrus**, Gray matter, Brodmann area 22, Range = 0	(65, −37, 5)	30	real/global
Left cerebrum, Occipital lobe, **Cuneus,** Gray matter, Brodmann area 18, Range = 0	(−5, −95, 14)	30	real/global
Left cerebrum, Frontal lobe, **Inferior frontal gyrus,** Gray matter, Brodmann area 44, Range = 2	(−49, 10, 19)	33	real/global
Left cerebrum, Temporal lobe, **Superior temporal gyrus**, Gray matter, Brodmann area 39, Range = 1	(−56, −62, 19)	33	real/global
Right cerebrum, Frontal lobe, **Middle frontal gyrus**, Gray matter, Brodmann area 9, Range = 0	(48, 14, 27)	18	real/global
Left cerebrum, Frontal lobe, **Superior frontal gyrus**, Gray matter, Brodmann area 8, Range = 0	(−2, 32, 44)	113	real/global
Left cerebrum, Frontal lobe, **Middle frontal gyrus,** Gray matter, Brodmann area 9, Range = 3	(−40, 10, 40)	67	real/global
Right cerebrum, Parietal lobe, **Inferior parietal lobule**, Gray matter, Brodmann area 40, Range = 0	(37, −39, 48)	175	real/global
Left cerebrum, Frontal lobe, **Middle frontal gyrus**, Gray matter, Brodmann area 6, Range = 0	(−38, −1, 49)	20	real/global
Left cerebrum, Frontal lobe, **Precentral gyrus**, Gray matter, Brodmann area 4, Range = 0	(−34, −24, 51)	30	ideal/local
Left cerebrum, Occipital lobe, **Middle occipital gyrus**, Gray matter, Brodmann area 19, Range = 5	(−27, −77, 12)	97	ideal/local
Right cerebrum, Occipital lobe, **Middle occipital gyrus,** Gray matter, Brodmann area 19, Range = 3	(31, −79, 11)	80	ideal/local

Location of center of mass includes output from Talairach Client set to search nearest gray matter to given coordinates.

**FIGURE 5:**
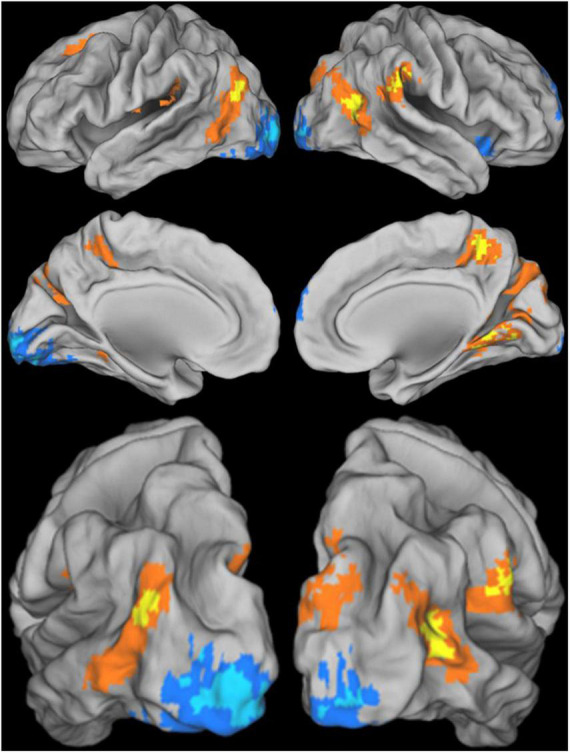
Conjunction analysis (see “5. Materials and methods”) of coaching and visual attention conditions shown from lateral, medial, and posterior-lateral views of the **left** and **right** hemispheres. Overlap of ideal (>real) self and global (>local) attention conditions are shown in warm colors (orange and yellow). Overlap of real (>ideal) self and local (>global) attention conditions are shown in cool colors (blue and cyan). Darker colors (orange and blue) indicate regions that pass a standard threshold for cognitive conjunction. Brighter colors (yellow and cyan) indicate regions that also pass a stricter threshold. Overlap incongruent to the direction hypothesized was minimal and is not shown. Statistical maps were computed in volume space then projected onto the PALS average surface atlas (Van Essen, 2005).

Figure 5 depicts overlapping brain regions in the two conditions for the entire brain. This figure shows that different brain areas are involved in resolving conflict in favor of ideal-self/global features as compared to real-self/local feature.

As shown in Figure 5, the real/local conjunction was present in the posterior occipital love (e.g., left inferior occipital gyrus). This region, which includes parts of the primary visual cortex corresponding to more foveal parts of the visual field, is responsible for initial processing of fine-grained local visual features. The ideal/global conjunction lay more anterior in occipital cortex, lying at the junction between occipital and temporal lobes (e.g., extending posterior from the end of the middle temporal gyrus). This region is known as a visual associative area involved in processing of high-level visual features. The ideal/global conjunction also recruited regions in medial parietal and temporo-parietal junction known to be involved in visual attention.

## 7. Discussion

We found that ICT coaching to the Ideal (and Accepted) self activates neural regions associated with resolving attentional conflict in favor of the global features of Navon figures, whereas coaching to the Real (and Ought) self activates neural regions associated with resolving attentional conflict in favor of local features. Based on the standard cognitive conjunction analysis, more than three times as much cortex showed overlap in the manner predicted (ideal/global and real/local) than in the other direction (ideal/local and real/global). Based on the statistically strict cognitive conjunctional analysis, we only found overlap in the manner predicted. The clearest differences between the conjunctions of ideal/global and real/local related to distinct cortical regions within each hemisphere. This finding is consistent with prior neuroimaging studies of global vs. local perception (Fink et al., 1996, 1997; Martinez et al., 1997; Han et al., 2002, 2004; Weissman and Woldorff, 2005). In addition, there was a tendency for ideal/global to show greater activity in the right hemisphere and real/local in the left hemisphere. This is also consistent with the prior neuroimaging findings cited and with neuropsychological research on global vs. local perception (Robertson et al., 1988; Lamb et al., 1989). The location of activation along the perceptual stream suggests that Ideal self coaching and global attention recruit neural regions in the visual associative cortex associated with imagination, whereas Real self coaching and local attention recruit areas associated with early sensory processing of component details (Ganis et al., 2004). Overlap included both occipital areas primarily involved in visual processing and parietal and temporo-parietal junction areas associated with attention and response selection (Corbetta and Shulman, 2002; Kubit and Jack, 2013). Importantly, the evidence suggests that functional differentiation of global vs. local processing is indicative of a high degree of conflict both for early visual areas (Hübner and Malinowski, 2002) and for higher areas involved in attention and response selection (Fink et al., 1996; Weissman and Woldorff, 2005).

It is notable that we found such extensive overlap between such disparate tasks. Our study design severely limits the psychological processes that can plausibly explain this overlap. In this regard, it is important to note that we were not looking for overlap in the main effects associated with the coaching and visual attention tasks. The coaching and visual attention tasks shared many features in common, such as visual perceptual and manual response demands. However, these cognitive components were identical or very similar for the paired tasks and so could not have influenced the contrasts used in the conjunction analyses. The visual attention task was highly decontextualized, containing no social or emotional cues of any sort. The conditions involved very similar stimuli and hence the differences in brain activity can only be plausibly attributed to attentional processes involved in resolving conflict in favor of global over local, and local over global representations, respectively. The coaching tasks were quite different from the visual attention tasks. They involved rich social cues that were contextualized by prior in person interactions with the two individuals that featured in the videos. On the other hand, there were no differences in visual perceptual demands between the two coaching conditions. The two coaching conditions involved video clips shot under identical conditions of the same two people (counterbalanced across participants for which condition they represented). That we nonetheless found extensive overlap both in early visual processing areas and in higher areas associated with visual attention provides strong support for ICT’s hypothesis that there is conflict between distinct aspects of the self and that attentional processes need to be recruited to resolve that conflict.

These findings support ICT’s contention that attentional processing is a highly relevant factor in determining how people respond to Ideal versus Real self-narratives (Boyatzis, 2008; Jack et al., 2013). This is significant because these links are likely to affect key coaching processes such as information processing and goal construal (Förster and Higgins, 2005). Global attention fosters creative, big picture thinking and integration of novel, uncertain, or incomplete data into inclusive, superordinate knowledge structures. Local attention, on the other hand, emphasizes differentiation, attention to detail, and activation of narrow cognitive categories that can lead to the omission of important incoming stimuli (Förster, 2012).

The current findings suggest that thinking about the self is like being presented with a multifaceted visual stimulus. Given that psychological accounts of the self generally hold that the self is constructed, it might seem reasonable to suppose we can think about whatever aspect of the self we choose without constraint or conflict. The current finds suggest a rather different picture. It appears that instead we are bombarded by a variety of self-referential thoughts that we must choose between. This picture fits well with evidence from studies of mind wandering, stimulus unrelated thoughts, and random experience sampling, all of which indicate that we have a tendency toward spontaneous self-referential thoughts even when our attention is primarily directed outside the self (Hurlburt and Heavey, 2006; Stawarczyk et al., 2011). It also fits with a key observation that guided the development of ICT: the biggest challenge to developing a vision of one’s ideal self are the multiple “ought selves” that others have imposed upon us and that we have internalized. It is precisely because these limiting thoughts are so readily available to us that ICT emphasizes the critical importance of a sustained and uncontaminated focus on the Ideal self at the beginning of a coaching engagement. This enables the coachee to break free from introjects to identify a path defined by their personal values. We are of course not the first to note the importance of this process–it is theme that emerges in the writings of Freud, Jung, Rogers, and others. Not only does a focus on the Ideal self help foster an increased attitude of acceptance toward the Real self, but it also produces greater sustained motivation to change by fostering intrinsic motivation (Taylor et al., 2019). Much as coachees may seek to conform to externally imposed expectations, activating the Ought self tends to trigger unconscious resistance or willfulness. This idea is captured by both the Gestalt Paradox of Change and by the central dialectic of Dialectical Behavior Therapy: the more we accept ourselves as we are, the more open we become to change. An important theoretical contribution of the current study is that it identifies a mechanism that explains this paradox or dialectic.

The most practically significant implications of these findings concern the sequencing and emphasis of different types of self-narrative in coaching conversations. The conflict between Ideal and Accepted and Real and Ought selves suggests that coaches should be highly intentional about when and how they engage discussions about different aspects of the self. It has been hypothesized that PEA coaching (i.e., a focus on vision, values and compassion) enhances a person’s energy and increases internal resources needed for change efforts. In contrast, NEA coaching (i.e., a focus on current problems and challenges or perceived needs to change as proposed by others in one’s environment) decreases, drains and depletes internal resources available for such effort.

The correspondence between Ideal versus Real self coaching and global versus local perception provides important clues to why it is more effective to coach first and foremost to the Ideal self. One reason for this relates to goal construal. Empirical work related to Regulatory Focus theory has already established a link between visual attention and promotion versus prevention focus (Förster and Higgins, 2005). Coaching to the Ideal self elicits a promotion focus (global) whereas coaching to the Real self elicits a prevention focus (local). A prevention focus may help an individual initiate goal-relevant action more quickly and decrease the likelihood of getting distracted by other things (Freitas et al., 2002). However, a promotion focus would be most effective at helping an individual adopt new goals, persist at them, and find enjoyment in striving toward them (Freitas and Higgins, 2002; Freitas et al., 2002; Sue-Chan et al., 2012).

A second reason why it is more effective to coach first and foremost to the Ideal rather than the Real self relates to positive versus negative emotion. When the Ideal self is activated, it is accompanied by affirming thoughts, a connection to that which is deeply meaningful, and a sense of optimism and self-efficacy that correspond to an increase in positive emotions (Howard, 2006). When the Real self is activated, it may be accompanied by self-conscious thought and fears of social evaluation, which give rise to negative emotional states. Because of a natural proclivity toward negative or threating information over positive information, exploration of the Real self tends to overshadow thoughts about the Ideal self, causing coachees to focus on deficits, stories of short-comings, and the need to comply with social expectations, pressures, and controls. Barbara Fredrickson’s broaden-and-build theory holds that positive emotions support the developmental process in a host of ways. Even fleeting experiences of positive emotions, such as joy, interest, contentment, and love, build an individual’s resources to respond effectively to more negative emotional experiences (Fredrickson, 2001). Positive affective states broaden our perceptions in a literal sense (Fredrickson and Branigan, 2005; Johnson et al., 2010) and metaphorically through an enhanced ability to see interconnections between disparate concepts, more inclusive cognitive categories, and enhanced memory and creativity (Isen, 1987; Fredrickson, 1998; Johnson et al., 2010). Positive emotions also facilitate persistence in learning to the point of mastery (Fredrickson, 1998). Positive emotions contribute to building social bonds and increase the likelihood of cooperation and reciprocity. Finally, positive emotions can serve as a buffer to chronic stress, providing support for behavioral, cognitive, and biological coping mechanisms (Fredrickson, 2001).

A third reason it is good to coach first and foremost to the Ideal self derives from Self-determination theory (SDT, Ryan and Deci, 2002; Deci et al., 2017). SDT distinguishes between two broad types of motivation. Extrinsic motivation is elicited by rewards and punishments. Intrinsic motivation arises out of the individual’s inherent desire to grow and fulfillment of the psychological needs for autonomy, relatedness, and competence. Intrinsic motivation is both more powerful and more sustained than extrinsic motivation. Furthermore, in at least some contexts, it has been shown that extrinsic motivators (rewards and punishments) can have the ironic effect of reducing intrinsic motivation (Sansone and Harackiewicz, 2000). In other words, while extrinsic rewards and punishments may produce short-term effects, they also sometimes undermine the only type of motivation that remains sustained without constant external reinforcement. ICT coaching evokes intrinsic motivation by helping the coachee fulfill the three key psychological needs of autonomy, relatedness, and competence (Taylor et al., 2019). By contrast, pressure to conform to organizational expectations is an external motivator because it is closely linked to rewards, such as promises of promotion and increased salary, and to punishments, such as negative performance reviews and threats to job security.

A fourth and final reason to adopt the ICT coaching sequence relates to Regulatory Focus Theory’s distinction between promotion and prevention motivation (Higgins, 1997). Promotion focused motivation is concerned with accomplishments, hopes, and aspirations; whereas prevention focused motivation is concerned with security, protection and prevention of negative outcomes. Regulatory Fit Theory (Freitas et al., 2002) extends Regulatory Focus Theory. It predicts that when people perceive a match or “fit” between orientation to a goal and the means used to approach the goal then that creates a feeling of “rightness” about the activity and increases engagement. Since coaching is a personal development activity, it fits promotion focus better than prevention focus. Hence, just like the alternative motivational theory of SDT, regulatory fit theory also predicts greater motivation and engagement in the change process when coaches encourage individuals to focus on the Ideal self. By contrast, the prevention focused motivation elicited by an initial focus on the Real self fits less well with coaching and other developmental activities, decreasing motivation and engagement.

It is important to note that while the alternative theories mentioned help explain why coaching to the Ideal self is more effective, they fall short of predicting or explaining the results presented here. All three theories (Regulatory Focus and Fit Theories, Self-Determination Theory, and Broaden and Build Theory) suggest greater sustained motivation to change when thinking about the Ideal self rather than the Real self, however, they also suggest that it would be most effective to simultaneously think about both Ideal and Real aspects of the self. This is different from ICT, which claims the two ways of thinking are in conflict. First, the Regulatory Focus Scale (Fellner et al., 2007), measures promotion and prevention focus using two independent subscales. Individuals can score high (or low) on both promotion and prevention motivation. We do not know of any evidence that scores on these two measures tend to be negatively correlated. Second, there is an abundance of evidence that extrinsic rewards succeed in motivating people – indeed this is a foundational and well tested assumption in the field of economics. While intrinsic and extrinsic motivators may compete in some specific contexts, they clearly do not always interfere. If that were true, then it would be impossible to find anyone who genuinely enjoys their paid job. Third, while positive emotions enjoy various advantages over negative emotions, there can be little doubt that negative emotions also play an important role. Further, we are not aware of any evidence that suggests positive and negative emotions cannot work together. Colloquially, the best way to motivate is often by a combination of carrot and stick. In conclusion, only ICT theory predicts conflict between Ideal and Real aspects of the self, and thus can explain the Gestalt Paradox of Change and the central dialectic of Dialectical Behavior Therapy.

A strong implication of the current findings is that coaches should begin a coaching engagement by focusing as much as possible on the Ideal self. This exploration should then be used to frame subsequent conversations about the Real self, rather than allowing externally imposed needs and objectives to frame consideration of the Real self. Coaches should also focus on developing the individual’s strengths rather than their weaknesses, and they should employ empathic listening. These strategies should increase acceptance of the Real self. This will minimize the unintended and counterproductive psychological processes associated with thinking about the Real and Ought self that result in resistance, denial, and willfulness. These processes include negative emotion, prevention focused motivation, extrinsic motivation, and interference with thinking about Ideal and Accepted aspects of the self. This recommendation differs markedly from some current approaches to coaching that recommend making a presenting problem or specific change goal the predominant context and focus of the coaching conversation.

### 7.1. Limitations and future research

Several limitations to our study should be noted. First, the temporal orientation of coaching to the ideal self versus coaching to the real self may play a role in our results. Coaching to the ideal self leans toward the future, but also engages reflection and discussion of people who have helped a person in the past and their present core values. The real self leans toward the present, but it also addresses anticipated problems in the future. Research examining the effect of imagining an event (e.g., locking a door) in the distant versus near future demonstrated that distant events were associated with higher-level construal (e.g., securing the house) whereas proximal events were associated with lower-level construal (e.g., putting the key in the lock; Trope, 2003). We attempted to control for temporal effects in the coaching task by including an equal number of statements that were focused on the past, present, and future in each coaching condition. Further research is needed to tease apart the temporal domain to determine if it has an effect on neural activation.

Second, there is evidence that the positive-global and negative-local association may be dispositional. Trait anxiety and depression have been associated with a local bias, whereas positive mood and optimism have been associated with a global bias and inversely related to a local bias (Basso et al., 1996; Derryberry and Reed, 1998). In the current study, individuals with high levels of depression, anxiety, and stress were excluded from participation. Future research may want to consider if and how these traits moderate the relationship between coaching and attention.

Third, it is widely noted that fMRI represents a measure of neural processing which is both indirect (e.g., dependent on the existence of a reliable vascular response to neural activity) and correlational (e.g., might reflect neural activity which is not necessary for completion of the given task). While both these limitations are present for all fMRI research, it is worth noting that experimental work establishes that fMRI is a highly reliable measure of neural activity (Logothetis et al., 2001), and convergent evidence from TMS and lesion studies presents little reason to suppose there is any widespread tendency for incidental neural activation to occur (Sakai et al., 2002).

Fourth, it might be objected that the logic of this study depends on what cognitive neuroscientists refer to as “reverse inference.” Reverse inference occurs when psychological processes are inferred from brain activity. As Russ Poldrack and others have influentially argued, many instances of reverse inference are invalid (Poldrack, 2006). A classic example is the reverse inference that activation of particular an area of cortex commonly referred to as the “anterior cingulate cortex”^2^ indicates the presence of “cognitive conflict” in a task. This inference was based on some influential early work that showed that this region is sensitive to the degree of cognitive conflict in tasks. The inference was made in many published papers partly because this cortical region is recruited by a very wide variety of different tasks (Van Overwalle, 2011). It was later shown the inference was invalid because several other cognitive processes also recruit the same region. Increases in physical and/or cognitive effort recruit the same brain areas even in the absence of cognitive conflict. In other words, there is a one-to-many mapping between brain activity and cognitive processes. Hence, it is not possible to infer which of the many cognitive processes were involved purely based on brain activity. Poldrack’s critique influenced some researchers to believe that all reverse inferences are invalid. This conclusion is both overly pessimistic and unwarranted (Jack et al., 2017). If all reverse inferences were invalid, it would be impossible to conclude anything about cognition based on brain activity. Given that the brain is the organ of cognition and fMRI has been shown to be a highly reliable method to determine the differential recruitment of different brain areas, such a pessimistic conclusion is hardly plausible. In practice, the reason reverse inferences are often invalid is that they assume an overly narrow characterization of the function of a given brain area. We know the brain is functionally specialized; however, an appropriate characterization of the function of most brain areas is far broader than the fine-grained characterizations that psychologists tend to arrive at when they hypothesize about the processes mediating between stimulus and response in specific task contexts. Reverse inferences can be valid provided that the characterization of the function of the brain areas is sufficiently broad. One way to arrive at such broad functional characterizations is through meta-analysis of studies involving many disparate tasks. Another is to use the logic of cognitive conjunction–the same experimental logic we use in this study. Cognitive conjunction allows researchers to identify areas of overlap in brain activity between different types of task. It lends itself to identifying cognitive commonalities rather than distinctions – lumping rather than a splitting. Our experimental design involves pairs of tasks that are highly cognitively distinct. This greatly limits what overlap in cognitive processing it is plausible to infer. Further, our inference does not concern itself with highly specific cognitive processes, but rather with attentional processes involved in resolving conflict. Attention is commonly viewed as one of the most fundamental and overarching processes involved in cognition. Additionally, here we use conjunction analysis to identify overlap in function in not just one comparison, but two: one associated with “big picture” thinking across visual perceptual and self-related cognitive domains, the other associated with more focused thinking across visual perceptual and self-related cognitive domains. We find it hard to imagine how to explain both cases of overlap except through the operation of attentional processes. Hence, there are good reasons to suppose the inference we make in this study does not suffer from the problems commonly associated with reverse inference. Our inference appears to offer the simplest explanation of the data. We nonetheless acknowledge that future experiments might succeed in identifying other factors capable of explaining the two instances (ideal/global and real/local) of substantial overlap in brain activity we observe in this study. We invite others to put forward and test hypotheses to this effect.

Fifth, the population studied was limited to young adults. It is possible that attentional competition between ideal and real aspects of the self is greater in this population than older populations. Indeed, Jung and Rogers might both be read as predicting a diminution in competition between ideal and real self with increasing psychological maturity. There are a couple of reasons this may occur. One possible reason is that as the individual matures and learns from experience, their real self comes to better approximate their ideal self. Another possible reason is that as the individual matures, they come to accept aspects of their real self they previously rejected. We hypothesize that ICT coaching brings about both these changes. We welcome future studies that empirically test these intriguing possibilities.

Sixth, it might be argued that a more convincing way to establish conflict between Ideal and Real selves would involve a behavioral test. We welcome this, however, we note there are difficulties that would make such an experiment challenging. Both broaden-and-build theory and regulatory focus theory have found support by measuring reaction time to Navon figures. This evidence provides excellent support for the claims that positive emotion and promotion focus are associated with global perception, whereas negative and emotion and prevention focus are associated with local perception. However, these findings do not provide evidence for the existence of conflict between positive and negative emotion, or conflict between promotion focus and prevention focus. The presence of an attentional bias toward one aspect of a real or imagined stimulus (or other mental construct such as the “self”) over another aspect need not entail those aspects are in conflict. Further, broaden-and-build theory does not claim any conflict between positive and negative emotion nor does regulatory focus theory claim conflict between promotion and prevention motivation. Even if it were shown that there was both speeding of response to global (local) features and slowing of response to local (global) features of a Navon figure relative to a baseline condition, this would still be insufficient. The reason is that active conflict between global and local features is inherent to the Navon figure, therefore any evidence for attentional competition might be due to the test stimulus used rather than conflict between the underlying psychological processes being assessed. A behavioral demonstration of conflict between different emotional or motivational states would require the use of test stimuli that do not themselves generate attentional competition. Given the difficulties inherent in carefully controlling social cognition, the need to have participants repeatedly elicit genuine thoughts about their Ideal and Real selves, and the large number of trials that would be required to reliably measure reaction time differences, these authors believe it would be very challenging to construct a behavioral test statistically sensitive enough to provide evidence for or against attentional competition between Ideal and Real selves. We would be glad to be proven wrong about this by enterprising researchers. Potentially the negative priming paradigm (Tipper, 1985) could be used as inspiration for such a design.

Seventh, some readers may be inclined to think that links between visual perception and aspects of the self are metaphorical at best. More specifically, the objection might be made that the conceptual gap between the psychological processes involved in personal development on the one hand, and the neural processes involved in visual perception on the other hand, is too great to be bridged by a single study. We agree that these links have traditionally been thought to be abstract and purely metaphorical, however, we point to several reasons for believing that these metaphorical links reflect concrete and real connections. First, some 30% of the human brain is involved in visual processing. It would be surprising and wasteful if evolution had not caused this extensive computational machinery to be co-opted to assist other types of cognitive processing. Second, our previous neuroimaging study showed that brain regions traditionally thought to be specialized for visual processing are also engaged during effective coaching. This reveals a concrete basis for the metaphorical links between personal development and visual perception. Third, a substantial body of work on embodied cognition (Foglia and Wilson, 2013) suggests that there is much less of divide between abstract cognitive processes and processes directly involved in perception and bodily control than has been traditionally supposed. Fourth, links between visual perception and personal development have been important since the beginnings of scientific psychology, as exemplified by Gestalt therapy (Perls et al., 1951). Fifth, as already cited, two other more recent psychological theories supported by behavioral experiments have established links between motivational and emotional processes and visual attention (Fredrickson and Branigan, 2005; Förster and Higgins, 2005; Johnson et al., 2010). Sixth, several other brain imaging studies have successfully examined links between neural processes and highly abstract, socially constructed, and/or philosophical ideas such as morality, cultural and political identity, and concepts of self (Moll et al., 2003; Zhu and Han, 2008; Kitayama and Park, 2010; Van Bavel and Pereira, 2018). In sum, it may appear there is a vast conceptual divide between visual perception and thoughts about the self, such that the links made in the current study appear stretched. However, several considerations as well as the striking extent of neural overlap revealed by the current study suggest this apparent divide is more illusory than real.

## 8. Conclusion

The current study used fMRI to test the hypothesis of conflict between thinking about Ideal and Real aspects of the self. We conducted a conjunction analysis to find evidence of overlap in brain activity between starkly different tasks. On the one hand, we used a coaching task to compare coaching focused on the Ideal self with coaching focused on the Real self. On the other hand, we used a visual attention task to assess brain areas associated with resolving attentional conflict generated by Navon figures in favor of either global or local perceptual features. We found substantial overlap between the two task contrasts. This provides further empirical support for intentional change theory’s (ICT) assertion that PEA coaching is associated with “big picture” global processing whereas NEA coaching is associated with more detail-oriented local processing. Furthermore, it supports ICT’s hypothesis that, similar to the global and local features of Navon figures, there is inherent conflict between Ideal and Accepted aspects of the self and Real and Ought aspects of the self. ICT not only asserts that there is attentional competition but also that an initial focus on the Ideal self facilitates behavior change whereas early consideration of the Real self inhibits behavior change. To help their clients effectively process feedback from co-workers and others, coaches need to foster greater acceptance of the Real self by (i) framing exploration of the Real self as being in service of the Ideal self and (ii) focusing on developing the individual’s strengths rather than fixing their weaknesses. We discuss three other psychological theories which provide further support that thinking about the Ideal self is more effective at producing behavior change. Individuals whose ideal self is salient are better able to scan the broad environment and perceive emerging themes. They experience more positive emotion, are more open to new ideas, and possess more sustained intrinsic motivation. If the current findings are further confirmed, organizations should be made aware of the likely costs of imposing organizational goals on the coaching process. Coaches will have greater success at engendering the positive change sought by organizations by accepting that the coaching process must be framed purely in terms of the individual’s personal vision of an ideal future, rather than framing the coaching process in terms of presenting problems. Coaching certification processes would also want to be changed to reflect this shift in emphasis early in the coaching process and in the rubric used to code recorded coaching sessions for determining certification levels.

## Data availability statement

The raw data supporting the conclusions of this article will be made available by the authors, without undue reservation.

## Ethics statement

The studies involving human participants were reviewed and approved by the Case Western Reserve Institutional Review Board. The participants provided their written informed consent to participate in this study. Written informed consent was obtained from the individual(s) for the publication of any identifiable images or data included in this article.

## Author contributions

AP led the data collection, analyzed and interpreted the behavioral data. AJ analyzed and interpreted the brain imaging data. AJ and AP wrote the manuscript. All authors contributed to the experimental design, theoretical framing, and editing of the manuscript.

## Footnotes

1 For an overview see https://vimeo.com/17012062

2 This label is misleading because the anterior cingulate cortex is a massive structure that can be subdivided into functionally distinct parts. Further, while the relevant cortical area overlaps part of the anterior cingulate it also involved paracingulate and medial prefrontal cortical areas.
